# Supplementary material for: Different levels of hypoglycemia in patients with type 2 diabetes, their achieved mean HbA1c vs. all-cause and cardiovascular mortality
Source: PLoS One. 2023 Jul 26;18(7):e0288360. doi: 10.1371/journal.pone.0288360 (PMC10370691; doi:10.1371/journal.pone.0288360)
Supplement: Attachment — Submitted filename: Response to the editor and the reviewers_20230615.docx [file pone.0288360.s002.docx]

**Response to the editor and the reviewers**

**Response to the editor**

1. Please ensure that your manuscript meets PLOS ONE's style requirements, including those for file naming.

Authors’ reply

We tried our best to revise the manuscript according to PLOS ONE's style requirements.

2. We noticed you have some minor occurrence of overlapping text with the following previous publication(s), which needs to be addressed:

1. 10.7717/peerj.14609 (shaded with blue)

2. <https://doi.org/10.1371/journal.pone.0272137> (shaded with yellow)

3. <https://doi.org/10.1016/j.amjmed.2011.07.011> (shaded with green)

In your revision ensure you cite all your sources (including your own works), and quote or rephrase any duplicated text outside the methods section. Further consideration is dependent on these concerns being addressed.

Authors’ reply

We used Microsoft Word to compare the current manuscript to our previous works with another manuscript, and to our best rephrase the duplicate text. We quoted all the works above mentioned (reference 14, 12, and 19 of the revised manuscript)

3. Your ethics statement should only appear in the Methods section of your manuscript. If your ethics statement is written in any section besides the Methods, please delete it from any other section.

Authors’ reply

We deleted the ethics statement apart from the Methods section (Please see the page 23, Lines 471-473 of the revised manuscript), and amended the ethics statement including the full name of the ethics committee/institutional review board(s) in methods section (Please see the page 5, Lines 116-118 of the revised manuscript).

4. In your Data Availability statement, you have not specified where the minimal data set underlying the results described in your manuscript can be found. PLOS defines a study's minimal data set as the underlying data used to reach the conclusions drawn in the manuscript and any additional data required to replicate the reported study findings in their entirety. All PLOS journals require that the minimal data set be made fully available. For more information about our data policy, please see <http://journals.plos.org/plosone/s/data-availability>.

Upon re-submitting your revised manuscript, please upload your study’s minimal underlying data set as either Supporting Information files or to a stable, public repository and include the relevant URLs, DOIs, or accession numbers within your revised cover letter. For a list of acceptable repositories, please see http://journals.plos.org/plosone/s/data-availability#loc-recommended-repositories. Any potentially identifying patient information must be fully anonymized.

Important: If there are ethical or legal restrictions to sharing your data publicly, please explain these restrictions in detail. Please see our guidelines for more information on what we consider unacceptable restrictions to publicly sharing data: http://journals.plos.org/plosone/s/data-availability#loc-unacceptable-data-access-restrictions. Note that it is not acceptable for the authors to be the sole named individuals responsible for ensuring data access.

We will update your Data Availability statement to reflect the information you provide in your cover letter.

Authors’ reply

The data sets analyzed during the current study are not publicly available because the restrictions are imposed by the Research Ethics Review Committee of Far Eastern Memorial Hospital. Although we cannot share the data publicly, the minimal datasets are available upon request from the Research Ethics Review Committee of Far Eastern Memorial Hospital repository (via: https://www.femh-irb.org/index.php/procedure). Application to the Research Ethics Review Committee of Far Eastern Memorial Hospital (contact via irb@mail.femh.org.tw, reference number 108008-F) is available for researchers.

**Response to the reviewer 1**

1. Seng-Wei Ooi et al. investigated the relationship between several levels of hypoglycemia and all-cause or cardiovascular mortality and between mean HbA1c levels and all-cause or cardiovascular mortality in 27,932 patients with T2D in Taiwan (2008-2018). They found that significant increased risk of all-cause mortality was associated with only most severe level 3 hypoglycemia levels in patients with T2D. In addition, T2D patients with mean HbA1c ≥ 9% manifested significant higher all-cause and cardiovascular mortality than T2D patients with mean HbA1c < 9%.

This study was well conducted and the observed results provided considerable clinical significance in management of patients with T2D.

Since serum lipid disorders are critical problem for the development of cardiovascular events and its mortality, in addition to LDL-C, data of triglycerides and HDL-C are needed for the assessment.

Authors’ reply

We added data of triglycerides and HDL-C (Please see Page 6, Lines 147-148, Page 11, Table 1, of the revised manuscript) We also re-analyzed all the regression models with addition of triglycerides and HDL-C, and the results are presented in Tables 2 and 3 (Please see Page 13-14, Table 2 and Page 16-17, Table 3 of the revised manuscript).

**The response to the reviewer 2**

1. Thanks authors for carried out this study . there are some concern which authors mentioned in limitation which is acceptable.

I would suggest author to bring more pathophysiological explanations about how hypoglycemic could increase the risk of mortality ; in discussion section only other's studies result compared , but needs more explanations

Authors’ reply

We have revised the discussion section with addition of plausible pathophysiological mechanisms about how hypoglycemic could increase the risk of mortality. (Page 21, Lines 429-438 of the revised manuscript)

**The response to the reviewer 3**

1. The paper showed the relationship between various levels of hypoglycemia and mean glycated hemoglobin A1c (HbA1c) achieved with all-cause and cardiovascular mortality in patients with T2D.They reveal that patients with level 3 hypoglycemia marginally increased all-cause mortality, but not cardiovascular mortality. Hypoglycemia in patients with higher mean HbA1c rather than low mean HbA1c were associated with an elevated risk of all-cause or cardiovascular mortality, which is of interest.
